# Harnessing the Power of Mobile Phone Technology: Screening and Identifying Autism Spectrum Disorder With Smartphone Apps

**DOI:** 10.7759/cureus.55004

**Published:** 2024-02-26

**Authors:** Kavita Reddy, Amar Taksande, Bibin Kurian

**Affiliations:** 1 Child Health Nursing, Srimati Radhikabai Meghe Memorial College of Nursing, Datta Meghe Institute of Higher Education and Research, Wardha, IND; 2 Pediatrics, Jawaharlal Nehru Medical College, Datta Meghe Institute of Higher Education & Research, Wardha, IND

**Keywords:** ethical considerations, accessibility, screening, early diagnosis, smartphone apps, autism spectrum disorder (asd)

## Abstract

Integrating smartphone applications into screening and identifying autism spectrum disorder (ASD) represents a promising and innovative frontier within healthcare. This forward-looking paper examines the current landscape of ASD screening apps, shedding light on their potential advantages and addressing and navigating significant challenges. One of the most compelling aspects of these apps lies in their potential to democratize access to ASD screening, effectively breaking down geographical barriers. By using the widespread availability of smartphones, these apps make it possible for individuals, caregivers, and healthcare providers to engage in early ASD screening from virtually anywhere. This accessibility is especially crucial in underserved areas or regions with limited access to specialized healthcare services. Moreover, these apps offer a degree of objectivity that traditional screening methods may need help to match. By relying on data-driven algorithms and machine learning, they can provide a more impartial assessment of a child's behavior, minimizing the potential for subjective bias. This objectivity, combined with the ability to monitor and assess a child's development over time, empowers caregivers with valuable insights into their child's progress. However, as with any technological advancement in healthcare, integrating smartphone apps for ASD screening is not without its share of ethical and privacy considerations. Ensuring informed consent is obtained, especially when collecting data from children, is complex and critical. Striking the right balance between collecting necessary data and protecting an individual's privacy requires careful thought and transparent communication. Additionally, the "digital divide" represents a challenge that needs to be acknowledged and addressed. Not all individuals and families have equal access to smartphones or the technological literacy required to use these apps effectively. This disparity in access must be considered when developing and implementing app-based screening solutions to prevent exacerbating existing healthcare inequalities. Nevertheless, the future of ASD screening apps holds significant promise. Advancements in technology, including integrating advanced sensors, wearables, augmented reality, and machine learning, can further enhance the accuracy and depth of screening. Interdisciplinary collaboration between researchers, developers, clinicians, and educators is crucial to ensure that these apps are effective, culturally sensitive, and user-friendly. Furthermore, integrating these apps into broader healthcare systems, including electronic health records and telehealth platforms, can streamline the screening process and enable a more seamless transition from screening to diagnosis and intervention.

## Introduction and background

Autism spectrum disorder (ASD) is a neurodevelopmental condition characterized by various symptoms, including difficulties in social interaction, communication challenges, and repetitive behaviors. It is a lifelong condition that affects individuals differently, with varying severity. ASD is a complex disorder with no known single cause, but it is believed to result from a combination of genetic and environmental factors [1,2]. ASD has become a topic of increasing concern worldwide due to its rising prevalence [2]. Early diagnosis and intervention are crucial for individuals with ASD to improve their long-term outcomes. However, diagnosing ASD can be challenging, often requiring specialized assessments conducted by trained professionals. This has led to delays in diagnosis and intervention, preventing individuals with ASD from receiving the timely support they need [3]. ASD has seen a significant increase in prevalence in recent years, making it one of the most common neurodevelopmental disorders. It is estimated that worldwide, about one in 100 children has autism. While the exact causes of this increase are still under investigation, it is clear that improved awareness, changes in diagnostic criteria, and increased access to healthcare services have played a role [4].

In recent years, mobile technology has revolutionized healthcare in numerous ways. Smartphones and mobile apps have become powerful tools for improving access to healthcare services, enhancing patient engagement, and streamlining various medical processes. These devices can now monitor vital signs, deliver personalized health information, and even assist in managing chronic conditions [5]. Mobile technology's potential in healthcare extends beyond these applications. It presents an opportunity to address healthcare disparities, especially in remote or underserved areas, by providing access to healthcare resources that were previously unavailable. The ubiquity of smartphones makes them a promising medium for reaching a broad range of individuals, including those who may be at risk for ASD or in need of early diagnosis [6].

The primary objective of this paper is to examine the innovative intersection of mobile technology and healthcare, specifically in the context of ASD. We aim to explore the potential of smartphone apps as tools for ASD screening and identification. This exploration encompasses an investigation into the existing landscape of smartphone apps designed for ASD screening and identification and an examination of the technologies and methodologies they deploy. We will also consider the challenges and limitations of this approach, including ethical considerations and the need for validation.

## Review

Understanding ASD

Definition and Characteristics of ASD

ASD is a complex neurodevelopmental condition characterized by various symptoms and behaviors. It is often called a "spectrum" because individuals with ASD can present with varying degrees of severity and a wide array of symptoms. Some common characteristics and features of ASD include:

Impaired social interaction: Individuals with ASD may have difficulty with social skills, such as making and maintaining eye contact, understanding and appropriately responding to social cues, and engaging in reciprocal conversations. They may struggle with understanding the perspectives and emotions of others, which can affect their ability to form and maintain relationships [7].

Communication challenges: Many individuals with ASD experience verbal and non-verbal communication difficulties. Some may have limited language abilities and struggle to express their thoughts and needs effectively. Others may have language skills but use language in unusual ways, such as echolalia (repeating words or phrases) or a lack of understanding of figurative language [7].

Repetitive behaviors: Repetitive actions or routines are often observed in individuals with ASD. These can include stereotypical behaviors like hand-flapping, rocking, or finger-flicking. Individuals with ASD may also adhere rigidly to specific routines and become distressed when these routines are disrupted. The insistence on sameness is a standard feature [8].

Restricted interests: Individuals with ASD may develop intense interests in specific topics or activities. These interests can be highly focused and specialized, often excluding other subjects. These intense interests can be a source of motivation and comfort for individuals with ASD [9].

Sensory sensitivities: Many people with ASD have heightened sensitivities to sensory stimuli, such as lights, sounds, textures, or tastes. They may experience sensory overload in response to specific sensory inputs, leading to discomfort or anxiety. Conversely, some individuals may seek sensory stimulation by repeatedly flapping their hands or tapping objects [10].

Difficulty with change: Routine changes or transitions can be challenging for individuals with ASD. They may become anxious or distressed when faced with unexpected alterations in their environment or daily schedule. Predictability and routine often provide security and comfort [11].

The Importance of Early Intervention

Improved communication skills: Early intervention programs often include speech therapy and communication-focused interventions. These interventions can help individuals with ASD develop better communication skills, including language comprehension, expression, and social communication. Improved communication can lead to increased social engagement and an enhanced quality of life [12].

Enhanced cognitive development: Early intervention strategies also focus on cognitive development. These programs aim to help individuals with ASD acquire essential cognitive skills, such as problem-solving, decision-making, and adaptive behavior. Early cognitive intervention can lay the foundation for improved learning and skill acquisition [13].

Better social integration: Social challenges are a hallmark of ASD. Early intervention can facilitate improved social interactions and integration with peers, family members, and the broader community. Interventions often target social skills, such as understanding social cues, initiating and maintaining conversations, and developing friendships [14].

Reduced behavioral challenges: Challenging behaviors are common in individuals with ASD and can significantly impact daily life. Early intervention strategies are designed to address these challenging behaviors, providing individuals with coping mechanisms and alternative ways to express their needs and emotions. Effective intervention can reduce the severity and frequency of challenging behaviors, making daily routines more manageable [15].

Support for families: Early intervention programs recognize the importance of supporting families. These programs provide families with guidance, education, and resources to understand better and assist their loved ones with ASD. Families learn effective strategies for communication, behavior management, and creating a supportive home environment. Early intervention can alleviate some stress and uncertainty families may experience with an ASD diagnosis [16].

Optimal developmental window: Early intervention capitalizes on the brain's neuroplasticity during the developmental years. Interventions delivered during this critical period can have a more significant and lasting impact on skill development and neural connectivity. The earlier intervention begins, the more effectively it can address core deficits associated with ASD [17].

Long-term outcomes: Early intervention can improve long-term outcomes for individuals with ASD. It can improve functional independence, increase opportunities for inclusion in mainstream settings, and reduce the need for specialized support as the individual progresses [18].

Mobile technology and healthcare

The Rise of Mobile Technology in Healthcare

Proliferation of smartphones: The widespread adoption of smartphones has empowered individuals to take greater control of their health. Virtually everyone now carries a powerful computing device with internet connectivity, allowing access to healthcare information and services on the go. Smartphones have become essential for health-related activities, from tracking fitness to monitoring chronic conditions [5].

Health and fitness apps: Health and fitness apps have become a staple of mobile technology. These apps enable users to track physical activity, monitor diet, manage chronic conditions, and set health-related goals. They have significantly promoted preventive healthcare and wellness by providing individuals with tools to monitor and improve their health behaviors [19].

Telemedicine: Mobile technology has facilitated the growth of telemedicine, which involves remote consultations with healthcare providers. Telehealth services have become especially crucial during public health crises like the COVID-19 pandemic, ensuring continuity of care while minimizing in-person interactions. Patients can access medical advice, prescriptions, and consultations from the comfort of their homes through mobile apps and video calls [20].

Remote patient monitoring: Mobile devices and apps can now collect and transmit real-time health data. This capability enables healthcare providers to remotely monitor patients with chronic conditions like diabetes, hypertension, or heart disease. Continuous data collection and analysis allow timely interventions, personalized treatment adjustments, and improved disease management. This approach has reduced hospital readmissions and improved patient outcomes [21].

Health information access: Mobile technology provides easy access to health information and resources. Mobile apps and websites offer reliable medical information, medication reminders, symptom trackers, and access to electronic health records. Patients can educate themselves about their conditions, treatment options, and preventive measures, empowering them to make informed healthcare decisions [5].

Wearable devices: The integration of healthcare sensors into wearable devices, such as fitness trackers and smartwatches, has further expanded the role of mobile technology in health monitoring. These devices collect data on physical activity, heart rate, and sleep patterns. Users can sync this data with mobile apps to comprehensively view their health and fitness [22].

Patient engagement: Mobile technology fosters greater patient engagement by providing tools for communication with healthcare providers, appointment scheduling, and medication management. Patients can actively participate in their care, improving adherence to treatment plans and health outcomes [23].

Benefits of Using Smartphone Apps in Healthcare

Accessibility: Healthcare apps are readily accessible to many users, regardless of location. This accessibility democratizes healthcare by breaking down geographical barriers. Patients in remote or underserved areas can access information, resources, and telehealth services through their smartphones, improving their healthcare access [24].

Personalization: Healthcare apps can provide personalized health information and recommendations based on user data. These apps can offer tailored advice and interventions by analyzing user-generated data, such as health metrics, symptoms, and preferences. Personalization enhances the relevance and effectiveness of healthcare interventions, leading to better health outcomes [25].

Engagement: Apps can engage users in health management by offering features like medication reminders, appointment notifications, and gamification elements. These engagement strategies motivate individuals to adhere to treatment plans, make healthier choices, and actively participate in healthcare. Increased engagement can lead to improved treatment outcomes [26].

Data collection: Mobile apps can collect extensive data on users' health and behavior, including vital signs, physical activity, and symptom progression. This data can be transmitted securely to healthcare providers, enabling them to make more informed decisions and interventions. Real-time data collection and remote monitoring are precious for chronic disease management and early detection of health issues [27].

Cost-efficiency: Mobile apps can be cost-effective compared to traditional healthcare delivery methods. They reduce the need for in-person visits, streamline administrative tasks, and optimize resource allocation. This cost-efficiency benefits individuals and healthcare systems, potentially lowering healthcare expenses and improving resource utilization [24].

Health education: Healthcare apps often include educational content, enabling users to access reliable health information and resources. This empowers individuals to make informed decisions about their health and well-being. Health education through apps promotes health literacy and encourages proactive health management [28].

Remote and continuous monitoring: Mobile apps enable remote monitoring of patient's health conditions. This is especially valuable for chronic disease management, post-operative care, and early intervention in critical health events. Remote monitoring reduces the burden on healthcare facilities and allows for timely interventions when health parameters deviate from normal ranges [29].

Previous Applications of Mobile Technology in ASD Management

Communication apps: Augmentative and alternative communication (AAC) apps, available on smartphones and tablets, have proven invaluable for individuals with ASD who may have communication challenges. These apps allow non-verbal or minimally verbal individuals to express themselves through symbols, pictures, or text-to-speech capabilities. Communication apps enhance the ability to convey needs, thoughts, and feelings, improving overall communication and reducing frustration [30].

Behavior tracking: Mobile apps have been used to track and analyze behavioral patterns in individuals with ASD. Caregivers, therapists, and educators can use these apps to record and monitor behaviors, identifying potential triggers and patterns. Data-driven insights from these apps assist in developing targeted interventions and personalized behavior management plans [31].

Visual schedules: Visual schedules and timers on mobile apps help individuals with ASD manage their daily routines and transitions. These apps use visual cues like pictures or icons to represent tasks and activities. Visual schedules provide predictability, reduce anxiety, and promote independence by guiding individuals through their daily routines step by step [32].

Social skills training: Mobile apps have been developed to teach and reinforce social skills to individuals with ASD. These apps often employ interactive games, videos, and scenarios to help users learn and practice essential social skills, such as recognizing emotions, understanding social cues, and engaging in appropriate social interactions. They provide a structured, engaging way to improve social competence [33].

Parent training: Mobile apps offer resources and training for parents and caregivers of individuals with ASD. These apps provide access to information, strategies, and support networks, empowering parents to understand better and address the unique needs of their loved ones. They may include guidance on behavioral interventions, communication strategies, and resources for navigating the challenges of ASD [34].

Smartphone apps for ASD screening

Overview of Existing Smartphone Apps for ASD Screening

ASD screeners: Several apps are designed based on established ASD screening tools, such as the Modified Checklist for Autism in Toddlers (M-CHAT) and the Social Communication Questionnaire (SCQ). These apps guide parents or caregivers through questions and prompts to assess a child's behavior and development. They follow structured questionnaires to identify potential signs of ASD, helping users gauge the risk and prompting further evaluation if necessary [35].

Data-driven apps: Some apps focus on longitudinal data collection, tracking various developmental milestones and behaviors over time. These apps rely on data analysis to identify patterns and trends that may indicate a heightened risk for ASD. By continuously monitoring a child's development, these apps can offer insights into potential concerns as they emerge [36].

Video analysis apps: A subset of apps enables users to record videos of children's behavior and activities. These recorded videos are then subject to computer algorithms and video analysis techniques. The algorithms are designed to detect potential signs of ASD, such as atypical eye contact, repetitive movements, or social interaction patterns. Video analysis apps offer a visual and objective means of assessing behavior [37].

Artificial intelligence (AI)-powered apps: AI and machine learning (ML) are increasingly being leveraged to develop apps recognizing patterns in speech, language, and behavior associated with ASD. These AI-powered apps aim to provide more objective assessments by analyzing a wide range of data, including vocalizations, language usage, and responses to questions. They excel at identifying subtle but consistent patterns that may not be readily apparent to human observers [38].

Features and Functionalities of Effective Screening Apps

User-friendly interface: User-friendliness is paramount. Apps should have an intuitive and user-friendly interface, making it easy for parents, caregivers, or professionals to navigate and use without extensive training. A simple interface ensures accessibility for a broad user base [39].

Comprehensive assessment: Effective screening apps should offer comprehensive assessments that cover a broad range of developmental domains associated with ASD. This includes evaluating social interaction, communication skills, and the presence of repetitive behaviors. A holistic assessment ensures that potential signs of ASD are thoroughly examined [40].

Age-appropriate content: ASD screening apps should be tailored to different age groups, accounting for developmental variations in children. Questions and assessments should be age-appropriate to ensure the screening process accurately reflects the child's developmental stage. This customization enhances the app's relevance and accuracy [41].

Data privacy and security: Ensuring the privacy and security of the data collected is paramount. Effective screening apps must implement robust data protection measures, including encryption, anonymization, and secure storage practices. Users should have confidence that their sensitive health information is safeguarded [42].

Integration with healthcare systems: Effective apps should be designed to integrate with healthcare systems seamlessly. This integration allows for a smooth transition from screening to diagnosis and intervention. It ensures that the data collected during screening can be shared securely with healthcare professionals, facilitating further assessment and support [24].

Scalability: Given the potential demand for early ASD screening, apps should be scalable to accommodate many users. Scalability ensures that the app can efficiently serve a broad user base, including parents, caregivers, and healthcare professionals, without performance issues or delays [41].

Feedback and recommendations: Effective screening apps should provide users with meaningful feedback based on the assessment results. If the app identifies potential signs of ASD or developmental concerns, it should offer recommendations for the next steps, including seeking further evaluation by healthcare professionals. This guidance empowers users to take informed actions based on the screening outcomes [31].

Case Studies of Successful ASD Screening Apps

Autism and Beyond (by Duke University): This pioneering research app utilizes ML algorithms to analyze facial expressions and vocalizations of young children to screen for ASD. By leveraging smartphone cameras and microphones, the app can detect subtle behavioral cues associated with ASD. Research studies have demonstrated its ability to identify potential signs of ASD accurately. The app not only contributes to early identification but also aids in the understanding of the behavioral markers of ASD [43].

M-CHAT-R/F (Modified Checklist for Autism in Toddlers, Revised with Follow-Up): The M-CHAT-R/F, adapted into app form, is a widely recognized and utilized tool for ASD screening in toddlers. This app provides a structured questionnaire that assesses a child's behavior and development. Parents and healthcare providers can use it to gain insights into a child's development and potential risk for ASD. The app's user-friendly interface and accessibility make it a valuable resource for early identification [44].

Cognoa: Cognoa is an innovative app that combines ML and video analysis to screen ASD. Parents can submit videos of their children's behavior and interactions, which are then analyzed by the app's algorithms. Cognoa has received recognition from the U.S. Food and Drug Administration (FDA) for its ability to identify potential signs of developmental delays, including ASD. The app's non-invasive approach and accessibility empower parents to actively monitor their child's development [45].

Proloquo2Go: While not a traditional screening app, Proloquo2Go is a powerful communication app designed to support individuals with ASD with communication challenges. The app provides a customizable grid of symbols and words that users can tap to generate speech output. It aids non-verbal or minimally verbal individuals in expressing their needs, thoughts, and feelings. Proloquo2Go is widely used as an augmentative and alternative communication (AAC) tool, significantly enhancing the communication abilities of individuals with ASD [46].

Identifying ASD with smartphone apps

The Role of AI and ML

Pattern recognition: AI and ML algorithms excel at analyzing vast amounts of data, including behavioral patterns, language usage, and responses to questions. These algorithms can identify subtle but consistent patterns associated with ASD, which may not be apparent to human observers. By recognizing these patterns, AI and ML contribute to more accurate and early identification [47].

Objective assessment: AI and ML provide a more objective assessment than traditional methods relying on subjective observations. This objectivity reduces the risk of bias in the identification process and improves the reliability of ASD screening. AI algorithms base their assessments on data-driven patterns and metrics, minimizing the influence of individual judgment [48].

Continuous learning: ML models can continuously learn and adapt based on new data. This adaptability allows for refining screening algorithms, enhancing their accuracy and effectiveness. As more data becomes available and researchers gain deeper insights into ASD, AI-powered apps can evolve to provide increasingly precise identification [49].

Scalability: AI-powered apps are well-suited to handle large data volumes, making them scalable to accommodate a broad user base. This scalability is crucial for widespread adoption and early identification efforts. AI algorithms can efficiently process data from numerous individuals, making it feasible to screen a large population for ASD risk [50].

Automation: AI and ML enable the automation of various aspects of the screening process. This automation reduces the burden on healthcare professionals and potentially expedites the identification of individuals at risk for ASD. AI algorithms can swiftly analyze data, generate assessments, and provide timely feedback, streamlining the screening workflow and facilitating prompt intervention when necessary [50].

Data Collection and Analysis Through Smartphone Apps

Data collection: Smartphone apps for ASD screening employ various data collection methods, including structured questionnaires, video recordings, and sensor data. These data capture multiple domains, such as social interaction, communication, and behavior. By collecting diverse data types, apps aim to comprehensively assess a child's developmental profile [31].

Real-time monitoring: Some apps are designed for continuous data collection over an extended period, allowing for real-time child development monitoring. This longitudinal approach enables the detection of trends and changes in behavior, which can indicate ASD risk. Real-time monitoring offers the advantage of early intervention by identifying potential concerns as they emerge [51].

Multimodal data: To paint a comprehensive picture of a child's behavior and development, apps may integrate data from multiple sources. This includes video recordings of social interactions, audio recordings of vocalizations, and sensor data (e.g., accelerometer data to assess motor skills). By combining these multimodal data streams, apps can offer a more nuanced and accurate assessment of an individual's ASD risk [52].

Data security: Ensuring the privacy and security of the data collected by these apps is paramount. Developers must implement robust encryption, data anonymization techniques, and secure storage practices to safeguard sensitive health information. Complying with data protection regulations and ethical standards is essential to maintain user trust and protect individuals' privacy [53].

Cloud integration: Many apps leverage cloud technology to securely store and analyze collected data. Cloud integration offers several advantages, including secure data backup, accessibility by authorized healthcare professionals and researchers, and the ability to perform advanced data analysis. Cloud-based solutions enable collaborative efforts in assessing and intervening in ASD cases, ensuring that the collected data serves a broader healthcare purpose [54].

Accuracy and Reliability of App-Based Identification

Validation studies: To establish the accuracy of app-based identification, extensive validation studies are indispensable. These studies involve rigorous comparisons of app-generated assessments with established clinical diagnostic criteria and expert evaluations. These validations are the foundation for gauging the app's ability to identify individuals with ASD accurately [55].

Sensitivity and specificity: App-based identification should demonstrate high sensitivity (the ability to identify individuals with ASD correctly) and specificity (the ability to identify individuals without ASD correctly). Achieving a balance between these two measures is essential to minimize false positives and negatives, ensuring that the screening results are accurate and reliable [56].

External validation: Independent validation by researchers and healthcare institutions is critical to verify the app's reliability and generalizability. External validation studies conducted by third-party experts help corroborate the accuracy of the app's identification process and reduce potential bias [57].

Regular updates: The landscape of ASD research and technology is continually evolving. Therefore, apps should be regularly updated to incorporate the latest research findings and improvements in AI and ML algorithms. These updates ensure that the identification process remains accurate and up-to-date, reflecting the most current knowledge in the field [48].

Transparency: Developers should provide transparency regarding the algorithms and methodologies used for identification. Openness about the app's inner workings allows users, caregivers, and healthcare professionals to understand and trust the app's results. Transparency fosters accountability and encourages ongoing scrutiny of the app's performance [58].

Ethical considerations: Ethical guidelines must be rigorously adhered to when collecting and using sensitive health data, particularly in the context of children. Ensuring informed consent from parents or guardians, as well as data protection, is paramount. Respecting privacy rights and ethical standards is fundamental to maintaining trust in app-based identification processes [59].

Clinical integration: While app-based identification holds promise as a screening tool, it should be viewed as a complementary component rather than a replacement for clinical evaluations by healthcare professionals. A confirmed diagnosis of ASD should involve a comprehensive assessment by a multidisciplinary team of experts, including pediatricians, psychologists, and speech-language pathologists. App-based identification can serve as a valuable initial screening step, facilitating early intervention and guiding individuals to the appropriate healthcare professionals for comprehensive evaluation and diagnosis [60].

Challenges and limitations

Ethical and Privacy Concerns

Informed consent: The ethical challenge of obtaining informed consent, particularly in the context of children, is a multifaceted issue. Parents or guardians must be fully informed about the data collection and sharing processes associated with smartphone apps used for ASD screening. This includes understanding how their child's data will be used, who will have access to it, and the potential implications for their child's privacy and future. Ensuring that informed consent is obtained, understood, and respected is essential to address this ethical concern. Moreover, obtaining the child's assent when age-appropriate is an additional consideration [61].

Data security: Protecting the security and privacy of sensitive health data collected by smartphone apps is paramount. Developers of these apps must prioritize robust data security measures, including encryption, secure data transmission, anonymization of data, and secure storage practices. Preventing data breaches and unauthorized access is essential to maintaining users' trust and protecting individuals' sensitive health information [62].

Data ownership: Determining the ownership of the data collected by these apps is a fundamental ethical question. Clear guidelines and policies should address data ownership, usage rights, and sharing permissions. Clarity helps users understand how their data will be used and who controls it. Balancing the interests of users, researchers, and developers in data ownership is a complex ethical consideration [63].

Data sharing and consent revocation: Users should be able to control how their data is shared and can revoke their consent at any time. This raises ethical questions about the feasibility of retroactively anonymizing and removing data once it has been collected and shared. Balancing users' control over their data with the practical challenges of data management and sharing is a crucial ethical dilemma that must be addressed [64].

Access to Technology and the Digital Divide

Socioeconomic disparities: The shift towards relying on smartphones and mobile apps for ASD screening can potentially exacerbate disparities in healthcare access. Families from lower socioeconomic backgrounds may face financial constraints that limit their ability to afford smartphones and data plans. This digital divide can create disparities in access to screening tools, preventing some individuals from benefiting from early detection and intervention. Addressing these socioeconomic disparities is crucial to ensuring equitable access to ASD screening [65].

Language and cultural barriers: Language and cultural differences can pose significant barriers to the effective use of smartphone apps for ASD screening. Apps must be accessible and culturally sensitive to diverse populations with varying linguistic backgrounds and cultural norms. This inclusivity is vital to ensure that all individuals can participate in screening programs regardless of their cultural or linguistic backgrounds [66].

Technological literacy: Using smartphone apps for screening effectively relies on users' technological literacy. Parents and caregivers, who play a central role in the screening process, must be comfortable using the technology and navigating the app's interface. Ensuring that apps are user-friendly and accompanied by clear instructions can mitigate technological literacy barriers. However, addressing this challenge requires educational efforts and support for those needing to be more tech-savvy [67].

Device compatibility: Not all families can access the latest smartphone models with the processing power and capabilities needed for sophisticated ASD screening apps. Older or less advanced smartphones may not support certain features or functions, potentially limiting the quality and accuracy of the screening process. Ensuring that apps are compatible with a range of devices, including older models, is essential to prevent disparities based on the quality of available technology [68].

Validation and Standardisation of App-Based Screening and Identification

Lack of standardization: One of the prominent challenges in app-based ASD screening is the absence of standardized procedures and protocols. The diversity of approaches and measures across different apps complicates comparing and validating these tools. The lack of common standards hinders efforts to assess their effectiveness consistently. Developing and adhering to standardized practices is crucial to ensure that app-based screening is reliable and accurate [41].

Variability in algorithms: The algorithms employed by different apps can exhibit significant variability, impacting the accuracy and reliability of screening results. Inconsistencies in algorithm development and implementation can lead to varying outcomes, making it challenging to trust the results across different apps. Establishing consistency in algorithm design, validation, and implementation is essential for building confidence in app-based screening [69].

Validation studies: Conducting comprehensive validation studies for app-based ASD screening is resource-intensive and time-consuming. Collaborative efforts involving developers, researchers, and healthcare institutions are necessary to execute rigorous validation studies effectively. The complexity of coordinating these efforts and the associated costs pose challenges, particularly when attempting to validate apps on a large scale. However, such studies are indispensable to establishing the credibility and reliability of screening tools [70].

Heterogeneity in ASD: ASD exhibits substantial heterogeneity, with individuals presenting various characteristics and challenges. Developing one-size-fits-all screening apps is challenging, as the tools must cater to different age groups, cultural backgrounds, and ASD profiles. Addressing this heterogeneity requires a nuanced approach, potentially leading to developing specialized apps tailored to specific subpopulations. Achieving this level of customization adds complexity to app development and validation [71].

Clinical integration: The successful integration of app-based screening into existing clinical workflows and healthcare systems presents logistical challenges. Ensuring that app-generated data seamlessly flows to healthcare providers and becomes part of the clinical decision-making process is essential. This integration necessitates clear communication channels, standardized data formats, and compatibility with electronic health records. Achieving this level of clinical integration is crucial for follow-up assessments and timely interventions [24].

Regulatory oversight: The absence of clear regulatory guidelines for app-based ASD screening and identification can lead to quality and safety standards variations. Developing appropriate regulations and oversight mechanisms is a complex endeavor that requires collaboration between app developers, researchers, and regulatory bodies. Striking the right balance between innovation and safety is paramount to ensure that app-based screening tools adhere to ethical and quality standards [60].

Future directions

Potential Advancements in Mobile Technology

Sensor technology: The future of mobile technology holds exciting prospects for enhanced ASD screening through advanced sensors. These sensors may be designed to detect and measure subtle physical and behavioral markers associated with ASD. For example, sensors could monitor physiological indicators such as heart rate, skin conductance, or movement patterns, providing valuable data for screening. Behavioral markers like vocal intonations and facial expressions could also be captured and analyzed. By incorporating such advanced sensors, future smartphones may significantly elevate the accuracy and depth of ASD screening, enabling a more comprehensive understanding of an individual's developmental profile [72].

AI and ML: The continuous evolution of AI and ML algorithms is poised to propel the development of more sophisticated app-based ASD screening tools. These algorithms are expected to become increasingly adept at recognizing nuanced patterns in data, including those indicative of ASD. By leveraging the power of AI and ML, these tools may enhance their predictive capabilities, potentially identifying ASD risk at an even earlier stage. This advancement improves screening accuracy and opens the door to more personalized and tailored interventions based on individual profiles [48].

Wearable devices: Wearable technology, such as smartwatches and fitness trackers, represents an intriguing avenue for integrating ASD screening into everyday life. These devices, equipped with various sensors, can continuously collect real-time physiological and behavioral metrics data. For instance, they can monitor heart rate variability, sleep patterns, physical activity, and stress levels. This wealth of information can offer insights into an individual's well-being and development, potentially aiding in the early identification of ASD. The seamless integration of wearable devices into screening processes can provide a holistic view of an individual's health and behavior, improving the overall screening experience [73].

Augmented reality (AR) and virtual reality (VR): AR and VR technologies can revolutionize the depth and accuracy of ASD assessments. These immersive technologies can create controlled and standardized assessment environments, allowing clinicians to observe and evaluate behavior in more realistic settings. For instance, VR scenarios can simulate social interactions, enabling professionals to assess an individual's responses and social skills. AR overlays can provide real-time feedback on eye contact and facial expressions during interactions. By harnessing AR and VR, future ASD screening tools may offer a more comprehensive and ecologically valid assessment of an individual's abilities and challenges [74].

Neuroimaging: Integration with portable neuroimaging devices is a frontier with the potential to revolutionize ASD screening. Devices such as EEG (electroencephalogram) headsets or smartphone-based eye-tracking technology can provide more direct measurements of brain activity and eye gaze patterns associated with ASD. EEG headsets can detect subtle electrical patterns in the brain, offering insights into cognitive processes and neural responses. Smartphone-based eye-tracking can analyze gaze behavior during social interactions, aiding in identifying atypical gaze patterns. Incorporating neuroimaging devices into ASD screening holds promise for unlocking deeper insights into the neurological underpinnings of ASD and refining early identification methods [75].

Collaboration Between Researchers, Developers, and Healthcare Professionals

Interdisciplinary teams: Encouraging collaboration among researchers, developers, clinicians, and educators is not merely advantageous but vital for the successful development of app-based ASD screening tools. These interdisciplinary teams bring together diverse perspectives, each contributing unique expertise. Researchers provide scientific rigor and a deep understanding of ASD, developers bring technical proficiency to create user-friendly apps, clinicians offer clinical insights and evaluation criteria, and educators provide valuable input on the educational and support aspects. This convergence of knowledge and skills ensures that the resulting screening tools are comprehensive, accurate, and tailored to the needs of individuals with ASD and their families [76].

Clinical validation: Close collaboration with healthcare professionals and researchers is the linchpin for the credibility and reliability of app-based ASD screening. Clinical validation studies conducted in partnership with clinicians are essential to confirm the accuracy and effectiveness of these tools. Healthcare professionals' insights into clinical practice, diagnostic criteria, and the intricacies of ASD play a pivotal role in refining the screening algorithms and validation protocols. By working hand in hand, researchers and clinicians can bridge the gap between technological innovation and clinical application [41].

User feedback: Involving individuals with ASD, parents, caregivers, and other end-users in the development process is integral to the success of these screening tools. Their experiences and feedback are invaluable for ensuring that apps are technically sound, user-friendly, and culturally sensitive. Gathering insights from those who will directly interact with these tools helps identify potential usability issues, cultural considerations, and areas for improvement. User feedback guides iterative development, resulting in more effective, inclusive, and empathetic screening solutions [77].

Research and development grants: Governments and organizations should recognize the significance of app-based ASD screening tools and provide funding and grants to support collaborative projects in this field. Research and development grants empower interdisciplinary teams to pursue ambitious initiatives, from the initial stages of tool development to clinical validation and beyond. These grants facilitate innovation, reduce financial barriers, and incentivize the development of cutting-edge screening tools that can ultimately benefit individuals with ASD globally [78].

Regulatory alignment: Collaboration between developers and regulatory agencies is pivotal in establishing clear guidelines and standards for app-based ASD screening. Regulatory alignment ensures that these tools meet stringent safety and effectiveness criteria, assuring users and healthcare professionals of their reliability. It also promotes transparency in app development, adherence to ethical standards, and responsible data handling practices. Collaborating with regulatory bodies fosters an environment where innovation and safety go hand in hand, instilling confidence in using app-based screening within the healthcare community [79].

The Integration of Smartphone Apps Into Healthcare Systems

Electronic health records (EHR) integration: The seamless integration of app-based screening results into EHR represents a critical advancement in healthcare. By incorporating the data generated by these apps into EHR systems, healthcare providers gain direct access to a wealth of information about a patient's developmental history and potential ASD risk factors. This integration streamlines the clinical assessment process, enabling healthcare professionals to make more informed decisions based on the collected data. It also facilitates communication and data sharing among healthcare providers, ensuring that all involved in the care of the individual with ASD have access to a comprehensive and up-to-date record [80].

Telehealth integration: The synergy between app-based screening and telehealth platforms holds immense potential for remote assessments and consultations. These integrated systems enable healthcare professionals to conduct assessments, consultations, and follow-up evaluations with individuals and families in the comfort of their homes. This is particularly valuable for individuals with ASD who may face challenges with in-person visits or reside in remote areas with limited access to specialized services. Integrating app-based screening into telehealth platforms not only enhances accessibility but also promotes continuity of care, allowing for timely interventions and support [24].

Data analytics and research: App-based screenings generate a wealth of aggregated and anonymized data. When harnessed effectively, this data can contribute significantly to large-scale research on ASD. Researchers can use these datasets to gain insights into the prevalence and characteristics of ASD in different populations, identify trends, and explore the effectiveness of various screening approaches. The integration of data analytics and research into healthcare systems not only advances our understanding of ASD but also informs evidence-based practices and interventions, ultimately benefiting individuals on the autism spectrum and their families [81].

Patient engagement: Integrating smartphone apps into patient engagement strategies fosters active participation by individuals and their families in their healthcare journey. These apps provide a platform for individuals to track their progress, access educational resources, and communicate with healthcare providers. Moreover, they empower families with a sense of agency, allowing them to actively contribute to their loved one's care plan. This engagement is essential for fostering a collaborative and patient-centered approach to ASD management, enhancing the overall quality of care [82].

Health system support: Successful integration of app-based screening requires support from healthcare systems, including staff training and infrastructure development. Healthcare institutions should invest in training professionals to effectively utilize these tools and interpret the data generated. Additionally, infrastructure enhancements may be necessary to securely accommodate the increased flow of digital health data. By providing this support, health systems can ensure that the benefits of app-based screening are fully realized, enhancing the efficiency and effectiveness of ASD diagnosis and intervention services [83].

## Conclusions

In conclusion, the intersection of smartphone apps and ASD screening holds immense promise in transforming the landscape of early diagnosis and intervention. The benefits are clear, with the potential to democratize access to screening, enhance objectivity, and empower individuals and their families with timely information and support. However, these advantages are accompanied by ethical considerations, privacy concerns, and the need for rigorous validation and standardization. The impact of app-based screening on individuals with ASD is profound, offering the prospect of earlier interventions and improved developmental outcomes. As we move forward, it is imperative to prioritize research and development, foster collaboration between stakeholders, and navigate these challenges with sensitivity and responsibility to harness the full potential of smartphone apps in the service of those on the autism spectrum and their families.
